# Inspectors’ responses to adolescents’ assessment of quality of care: a case study on involving adolescents in inspections

**DOI:** 10.1186/s12913-018-2998-9

**Published:** 2018-04-02

**Authors:** Suzanne Rutz, Hester van de Bovenkamp, Simone Buitendijk, Paul Robben, Antoinette de Bont

**Affiliations:** 1 Erasmus School of Health Policy & Management, Erasmus University Rotterdam, PO Box 1738, 3000 DR Rotterdam, The Netherlands; 2 Joint Inspectorate Social Domain, Utrecht, The Netherlands; 3 Health and Youth Care Inspectorate, Utrecht, The Netherlands; 4 Imperial College London, London, UK

**Keywords:** Quality of care, Participation, Inspectorates, Regulation

## Abstract

**Background:**

Users of care services are increasingly participating in inspections of the quality of care. In practice, incorporating service users’ views is difficult, as users may have other views on good care than inspectors and thus give information that does not fit the inspectors’ assessment criteria. This study compared the views on good care of young care users (adolescents) and inspectors, seeking to understand what the differences and similarities mean to incorporating the users’ views in inspections.

**Methods:**

We conducted a single-case study combining document analysis with a meeting with inspectors. The selected case came from a Dutch inspectorate and involved a thematic inspection of care for children growing up poor.

**Results:**

Inspectors and adolescents agree on the importance of timely care, creating opportunities for personal development, and a respectful relationship. The views on quality of care differ with regard to sharing information, creating solutions, and the right moment to offer help. We identified three ways inspectors deal with the differences: 1) prioritize their own views, 2) pass the problem onto others to solve, and 3) separate the differing perspectives. With similar viewpoints, inspectors use the adolescents’ views to support their assessments. When viewpoints conflict, information from adolescents does not affect the inspectors’ judgments. Explanations are related to the vulnerability of the adolescents involved, the inspectorate’s organizational rules and routines and the external regulatory context.

**Conclusions:**

Service user involvement in inspections potentially impacts the quality of care. Yet, conflicts between the views of service users and inspectors are not easily overcome in the regulatory context.

## Background

The ideal of active citizenship has gained ground in many Western countries [1–5]. One way people can exercise active citizenship in health and social care is to voice their preferences and experiential expertise so that services can be improved [5, 6]. With this aim, service users are increasingly invited to participate in decision-making processes on quality improvement, medical guideline development, government policymaking and inspections [2, 5, 7–12].

Despite its ideological appeal, research shows that involving service users is not easy to realize [2, 7, 10, 11, 13]. In the literature, difficulties are ascribed either to the service users who participate or the organizations that invite their users to participate. Studies show that participants are often no ‘ordinary’ service users [12]. They need specific skills and knowledge to participate successfully. Training service users to gain the skills and knowledge to wield influence is often brought up as a solution to foster their involvement [2, 12, 14]. While professionalization processes enable participation, they relegate service users’ experiential expertise to the background, consequently triggering discussions about participant representativeness [2, 3, 11, 14, 15]. On the side of organizations involving participants, the way that participation is arranged and the space provided for users’ input are regarded as barriers or resources for participation. Formal rules and bureaucratic routines in decision-making processes can, for instance, pose a challenge for participants, whereas a non-hierarchical organizational culture can be a resource for successful involvement [6, 11, 12, 14].

The rationale for service users’ involvement is based on the assumption that they have a distinct perspective which offers new options to improve the quality of services and strengthens decision-making [9, 10, 16, 17]. Consequently, the users’ perspective may conflict with organizational rules and conventions, and professional or societal standards such as safety and cost containment [11, 12, 18]. Hence, the key questions are whether such conflicts are addressed and, if so, how they are dealt with.

In this paper, we aim to advance our understanding of service user involvement in practice by taking the last issue as a starting point. We focus on service user involvement in one inspectorates’ assessment of quality of care. Inspectorates are expected to exercise control over care quality and protect vulnerable people from harm [19]. In many countries, user involvement is high on the inspectorate agenda [7, 16, 19–22]. In their regulatory work inspectors include all kinds of service users as lay inspectors, ‘mystery guests’ [16], through consultation or via analysis of social media [23] and complaints [20]. Although various forms of service user involvement in inspections have been studied [7, 13, 16, 20, 23–25], how inspectors use the perspective of services users and their input is underexplored.

We analyze how inspectors from the Joint Inspectorate Social Domain (JISD) involved the perspectives of young care users in an inspection of a broad range of social and health care services that provide help for children growing up poor in the Netherlands. The following questions guide our paper: *What do adolescents who have received care consider to be good care and how do their views compare to the assessment criteria inspectors use to evaluate care? How do inspectors deal with the similarities and differences to their own views and what can explain the inspectors’ ways of dealing?*

The next section describes the setting of the study and the methods used to answer the research questions. Our comparative analysis of adolescents’ views and the inspection criteria is at the heart of the paper. Finally, we discuss our findings, explain the inspectors’ ways of dealing with similarities and differences and relate these to difficulties described above in connection with participants and organizational contexts.

## Methods

### Setting of the study

The JISD is a partnership of four government inspectorates in the Netherlands: the Health and Youth Care Inspectorate, Inspectorate of Education, Inspectorate for Justice and Safety, and Inspectorate of Social Affairs and Employment (before 2017 this partnership was known as the Joint Inspectorate for Youth). Since its foundation in 2003, the JISD has included adolescents in inspections. Adolescents come along on inspections in the role of lay inspectors [7, 26], and inspectors hold consultative meetings with adolescents (including an interactive voting system), interviews and focus groups. JISD inspections are mainly theme-based and concentrate on public problems that cannot be solved by one organization or sector. Hence, inspections follow a multi-agency approach, including a broad range of local services through all sectors, such as health, youth care, education, police, and social affairs [27]. Examples of public problems subjected to thematic inspections are: child abuse, obesity, youth offending, addiction and poverty. In this study, we focus on the latter.

Regulatory work is divided into three phases: 1) gathering information about the service under scrutiny, 2) assessing whether the service complies with a set of assessment criteria, and 3) taking enforcement action for non-compliance to meet the criteria and make improvements [28–31]. Service users are increasingly included in information gathering (phase 1); the assumption is that they can provide useful signals and quality information, which may improve inspectors’ assessments [7, 20]. During the assessment (phase 2), inspectors evaluate whether the services under scrutiny ensure their users’ involvement as part of providing good care. This way, user involvement by services becomes part of the inspectors’ assessment criteria. In enforcement (phase 3), although inspectors consider the consequences of non-compliance by services for clients and patients [32, 33], the perspective of service users is often relatively implicit.

During the inspection of care for children growing up poor, the JISD inspectors included adolescents living in poverty in the information gathering phase (phase 1). They conducted interviews and focus groups, assisted by Stichting Alexander, a foundation specialized in youth involvement. Inspectors and workers from Stichting Alexander identified the organizations and stakeholders (eg, food banks, charities, youth workers and social workers) involved in services for poor families in four municipalities. They asked stakeholders to invite young people to take part in an interview or focus group. In practice, many young people were recruited by youth workers and the interview panels took place at community centres and youth clubs in poor neighbourhoods, where young men are better represented than young women (this is possibly why few young women took part in the inspection). The adolescents who did participate varied in other characteristics (eg, age and ethnicity; see Table 1).

**Table 1:** Demographic information on the adolescents that were involved in the inspection

Method	Interviews (n = 2) Focus groups (n = 10)
Number of respondents	43
Gender	Male (*n* = 37) Female (*n* = 5)
Age	10 (n = 1) 11 (n = 2) 12 (n = 2) 13 (n = 4) 14 (n = 6) 15 (n = 6) 16 (n = 7) 17 (n = 9) 18 (n = 4) 19 (n = 1)
Ethnicity	Dutch (n = 9) Moroccan (n = 9) Antillean (n = 6) Roma (n = 3) Surinam (n = 1) Afghan (n = 1) Iran (n = 1) Unknown (n = 12)

The inspectors and workers from Stichting Alexander conducted the interviews and focus groups following a semi-structured format. Topics included: what young people considered poverty, how they experienced their situation, whether they had received care and assistance, how they experienced this and what they considered to be necessary improvements for young people living in poverty. The interviews and focus groups were audio recorded and transcribed verbatim. The inspectors analyzed the reports of the interviews and discussed their analysis in an assessment meeting, where the information gathered via other inspection methods was also discussed.

### Study design

We used a single-case study design [34], selecting a case of thematic inspection of care for children growing up poor. Poverty is an ambiguous public problem. What the problem means for those affected, whether action should be taken and if so what action, is controversial [35]. This ambiguous subject is an excellent case to study how divergent viewpoints are dealt with, as adolescents living in impoverished conditions may have other views on good care than inspectors, and thus give information that does not fit the inspectors’ assessment criteria.

In terms of inspection, the case can be considered typical as it is conducted like any other inspection, following the three phases of information gathering, assessment and enforcement action.

### Data collection

We used multiple methods to study our case. Our data consisted of material created and used by inspectors during information gathering and assessment of the inspection (in total 68 documents) and a meeting with inspectors.

First, we collected the documents that inspectors prepared for the inspection to gain insight into the context and their decisions on methods and procedures. This material included the inspection plan, the set-up for interviews and focus groups with adolescents, the information given to them, minutes of the inspectors’ meetings, and inspection formats.

Second, we collected documents containing the information that the adolescents, who grew up poor, gave to inspectors during information gathering. This consisted of verbatim transcripts of the two interviews and ten focus groups the inspectors held and the inspectors’ notes that included their reflections on the interviews and focus groups (see also Setting of the study).

Third, we collected documents that inspectors created for their assessments and to communicate their decisions, material on the inspection framework and assessment criteria, evaluation reports of the information obtained from adolescents, and the inspectors’ reports that communicated the judgments.

Fourth, we held a meeting at the JISD to discuss the preliminary findings and explanations of our findings. The minutes of the meeting were added to the data collection.

### Data analysis

Our analysis focused on what the adolescents regarded as good care. Interview and focus group transcripts were read closely several times and coded inductively, locating recurrent subthemes and grouping subthemes together in themes [36]. We identified the following themes on quality of care: 1) trustworthiness and loyalty of professionals (eg, respectful relationship), 2) adolescents’ influence in the care process (eg, deciding when to ask for help), 3) use of information on adolescents’ situations (eg, privacy), 4) results of the care for adolescents (eg, offering practical solutions and timeliness), 5) creating opportunities for personal development (eg, finding a suitable internship, job or education). Ongoing analysis refined the specifics of each theme. Next, we analyzed the documents that inspectors produced during their inspection and compared the content with the five themes identified, based on the adolescents’ information. In this comparison we found three striking similarities and three fundamental differences. We analysed how inspectors dealt with the similarities and the differences and tried to explain their ways of dealing.

We had access to this data since the first author is also a JISD inspector. A disadvantage of this dual role is that it raises the issue of methodological distance. We managed this potential tension in two ways [37]. First, three authors analyzed the data. Two were outsiders to a regulatory context, which enabled the research team to question each other’s interpretations, stimulate self-reflection and challenge locally situated taken-for-granted notions. Secondly, we presented and discussed our preliminary findings at two conferences for researchers and a conference for inspectors of various Dutch inspectorates (excluding the JISD). These meetings helped us to enhance the reliability and validity of the analysis. Although both audiences recognized our findings, they held very different views on the implications. These different views helped us to look more thoroughly into the specifics of the regulatory context in order to find explanations in this context and to come up with suggestions for improvement.

### Ethical considerations

The adolescents (and their parents when the adolescent was younger than 16) all gave the inspectors their written informed consent to participate, with anonymity guaranteed. Consequently, we use pseudonyms for respondents’ names. According to the Dutch act on ‘Medical Research involving Human Subjects’, this type of research does not require the consent of an ethics committee as our study did not involve a medical intervention [38].

## Results

This section first describes the similarities between the views of adolescents and inspectors. Then it describes the differences that led to tensions in the inspection process and the ways the inspectors dealt with these tensions.

### Similarities: Timeliness, creating opportunities for adolescents to develop and a respectful relationship

We found three similarities in views. The first concerns timeliness of the care. The adolescents’ views on receiving care were rather negative; they voiced many complaints about the time it took to get results. Chantal for instance remarking on the care she received, said ‘*It all takes way too long*’ (G2). Ahmed, who does not go to school, agreed:

*Ahmed: It all goes way too slow for me. I’ve told them I want to go back to school, but they don’t do a thing.*

*Interviewer: So, are they looking for a new school for you? […].*

*Ahmed: Yes, they said that they’d arrange it within six weeks, but that was eight weeks ago (S5).*

For the inspectors as well, arranging services in a timely manner was important. In fact, it was one of their assessment criteria (criterion 4, Table 2). The second similarity concerned the perceived need to stimulate young people’s participation in society. Adolescents, like Ahmed, placed great value on schooling and stressed that they wanted professional help to find a suitable internship, job or education; another assessment criterion (criterion 5, Table 2). The third similarity was that both adolescents and inspectors valued a respectful relationship between the young person and care professional; stimulating this was also an inspection criterion (criterion 1, Table 2). For adolescents, a respectful relationship meant trust. It indicated that their views were taken seriously, that professionals kept their promises, did not discriminate, and showed respect for the adolescent’s choices even if they did not agree with them. For instance, commenting on his relations with professionals, Mateo said:

*We should be treated with a bit of respect. If professionals forbid everything and say ‘the way you do things is bad, and how I do them is good’, then they offend people. (S1).*

**Table 2:** The inspection framework to assess the quality of services for poor children and their families

Category	Inspection criteria
I. Tailoring services to young people’s needs	1. Organizations make sure their services fit the wishes and abilities of young people and their families and encourage forming good relationships 2. Professionals jointly analyze the situation of young people and their families and all the conditions that affect their lives, including underlying problems and causes 3. Services are tailored to the problems of young people and their families, and are aimed at preventing or solving the problems and underlying causes 4. Tailored services are arranged quickly
II. Participation and coverage rates of services	5. Professionals stimulate the active participation of young people and their families in society 6. Obstacles to receiving the services they need are removed for young people and their families 7. Organizations know which target groups do not use the services and reach out to these groups
III. Consistency of activities	8. Professional activities are aligned with organizational strategy 9. Various partners cooperate to achieve their goals efficiently. Their services are aligned and coordinated by one of the professionals 10. Professionals collect, record and exchange necessary information about a young person or family

When adolescents and inspectors held similar views, inspectors used the information gained from adolescents to support their findings. They quoted adolescents in their reports to illustrate their conclusions. For instance, one report quoted Mateo and went on to state:

*Young people emphasize that a good relationship is fundamental to providing good care. An important part of a good relationship is that a professional shows respect for a young person.* [39]

In the meeting with inspectors, the inspectors explained that illustrative quotes are important as they call on the services under scrutiny. For the service providers it conveyed a sense of urgency to act when their ‘own’ users talked about how issues in the care affected them [40]. In the assessment phase of the inspection, such quotes and examples of adolescents’ situations helped inspectors to convince the service providers of the value of the inspectors’ judgments and the action needed to make improvements.

### Differences: Sharing information, creating solutions and the moment to offer help and assistance

We found three differences in views that led to tensions in the inspection process.

#### Tension 1: Privacy versus sharing information

While inspectors and adolescents both found a respectful relationship with professionals an important aspect of good care, their perspective on what a respectful relationship entailed differed in key aspects. This led to the first tension we identify here. According to adolescents, an important element of a trustworthy relationship was that professionals did not share information freely with others. On the other hand, inspectors emphasized that professionals should exchange information.

For adolescents it was crucial to control who obtained specific information about their situation. Professionals who shared information with other professionals, without asking permission, lost their trust. The adolescents explained that they put new professionals to the test and would not give confidential information on first contact. Daniel, for instance, described what he did when he discovered that his social worker had discussed his situation with one of his teachers:

*Daniel: If I’d told her everything, she would’ve told my mentor and he would’ve talked about it all to the team leader, and then everything would have gone round. [...]*

*Interviewer: So what did you do then?*

*Daniel: Yeah, well fuck her, you know. She tries to make new appointments, but I don’t bother showing up now I know I can’t trust her. […].*

*Winston: I wouldn’t have talked to her in the first place.*

*Gabriel: I’d rather go to my parents if I’m in trouble. (C1).*

Sharing information without asking the adolescent’s permission resulted in distrust and avoidance of professionals. Yet, inspectors felt that professionals needed a complete understanding of adolescents’ situation to provide good services. Inspectors reasoned that to gain a complete understanding professionals needed to collect and exchange information about the young person’s situation to tackle the causes and intervene as early as possible (criteria 2 and 10, Table 2; see also tension 2). Inspectors dealt with the tension of respecting privacy and sharing information by subordinating adolescents’ views and emphasizing that professionals needed to share information. One of their reports stated:

*Although young people cherish their privacy, exchanging information is important for early intervention. It would be important to discuss this problem with young people to find out what solutions they can offer.* [41]

In the meeting, inspectors explained that they had important reasons for prioritizing their view. They felt these adolescents were members of a vulnerable group that lacked the ability to protect themselves and needed protection. Although they acknowledged what the adolescents thought, they gave their own view more weight. According to the inspectors, professionals needed to share information to identify situations in which vulnerable young people needed care and to enable interventions as early as possible. Inspectors reasoned that providing help early, before young people needed it urgently, stopped problems from exacerbating (see criteria 3 and 7, Table 2).

However, as Daniel’s quote illustrated, adolescents stated that the distrusted professionals who shared information, and consequently would not provide new information. Sharing information was then counterproductive to gaining a complete view of the adolescents’ lives. Yet, the inspectors felt that sharing information about an adolescent would not automatically result in distrust between adolescents and professionals. As stated in the quote above, the inspectors found it ‘*important to discuss this problem with young people to find out what solutions they can offer’.* During the meeting inspectors pointed to professionals to start this dialogue. They explained that exchanging information was key and that they expected professionals to be able to maintain a trustful relationship with adolescents and share information at the same time. This signified another way of dealing with this tension, namely: passing the dilemma on to others. Inspectors did not ease the tension themselves, but asked others to do it for them.

#### Tension 2: Finding solutions versus finding hidden problems

The second tension was that adolescents felt that professionals needed to focus on actively finding practical solutions for the problems they presented, while inspectors wanted professionals to look for causes and hidden problems, which adolescents associated with simply talking about problems, and not with solving them. For instance Mehmet remarked: ‘*Everything they tell you, you can also tell yourself’ (S4)*. Adolescents did not want to talk about causes, things that had happened in the past or other problems:

*Romario: Professionals just talk in circles. I don’t want people to talk to me so much. Sometimes I think they only talk about everything that happened to you in the past. And why you can’t change. (C2).*

According to the adolescents, talking was only effective when communication was part of the problem. For instance, Dave was very positive about a psychologist who mainly talked to help members of his family improve their communication: ‘*And as a result, now we all communicate smoothly [in our family].’ (G1).* Adolescents expected professionals to produce tangible results, offering practical solutions to the problems they wanted to solve, not necessarily all of their problems (including underlying causes). The problems could be about communication but also about other issues; they expected professionals to help them clear their debts, for instance or (as in the Ahmed case above) make arrangements so that they could get an education tailored to their wants and needs. Though the inspectors agreed on the importance of obtaining results (see also subsection on similarities), such as adolescents going back to school, they assumed that it was necessary to talk about the problem first, instead of focusing immediately on solutions. According to their assessment criteria, inspectors thought that professionals should jointly analyze a problem and reach consensus on its importance and causes to find the appropriate solutions (see criteria 2 and 3, Table 2). According to the inspectors, the fact that professionals did not conduct such an analysis was an important obstacle to the provision of good care. The inspectors’ report described this as follows:

*The care often starts late, after problems have become severe. Only short-term help is provided to tackle the problems, and professionals fail to deal with the causes. Among other reasons, this is because professionals do not analyze the whole problem in context when the care process starts. They often lack vital information on the family situation. Because they do not deal with the causes, there is a high chance that severe problems will recur* [42]*.*

The inspectors assumed that without problem analysis, the help provided would not address the underlying causes, which would lead to a recurrence of the problem. This matched the assumption that stopping problems from getting worse was important, as we explained above. Although adolescents and inspectors both found quick results for young people important, they differed in the method of obtaining results; inspectors take the problem as the starting point, not the possible solutions.

Inspectors dealt with the tension of finding rapid solutions versus first identifying hidden problems by describing the adolescents’ views and their own differing viewpoints in separate sections of the inspection reports. All inspection reports contained a chapter entitled ‘Living in poverty’, which described the perspective of adolescents in poverty, the consequences of growing up poor and the care required in this situation. In a subsection of the chapter entitled ‘Tailoring services to young people’s needs’ the inspectors reported the adolescents’ views. For example: *‘Young people and their parents expect the care process to start quickly, and, that the care is concrete and practical from the start. From their perspective, only talking does not help.’* [43] Another subsection of the same chapter, reflecting on the inspectors’ perspective, reported that professionals needed to analyze underlying causes and hidden problems. In other words, the tension was rendered invisible by separating the conflicting perspectives in different parts of the report.

#### Tension 3: Care for urgent matters versus early intervention

The third tension was that adolescents only seek help when they cannot solve the problem by themselves or with their families, whereas for inspectors it was important that professionals reached out to young people and solved problems at an early stage. Above, in Daniel, Winston and Gabriel’s discussion on respecting privacy, the boys agreed that they preferred to solve problems on their own or with relatives, rather than contacting a professional. Asking for help was a big step for them, which they did only for urgent matters that they really could not resolve. However, from the inspectors’ view of prevention, it was important that professionals reached out to adolescents and families while the problems were still small:

*Professionals [do not view] various groups of people as potential clients. For instance, this applies to the working poor and to people with relatively small problems. Care and assistance are offered to these groups less often. For example, professionals are less inclined to offer families with small debts (below 9000 euro) help than families with larger debts. However, these groups are vulnerable because a small adversity may trigger the development of severe problems. Therefore, from the viewpoint of prevention, help for the group with small problems is essential.* [42]

According to inspectors the group with small problems had special needs and was eligible for early intervention. Similar to the first tension about respecting privacy and sharing information, inspectors dealt with this third tension by giving their own view more weight. Inspectors attributed adolescents asking for help only for urgent matters to the bad experiences that many of these adolescents had had with care, which set up a negative cycle of aversion to contacting a caregiver again. They felt that adolescents would be more positive about early intervention if they had had more positive experiences in receiving care. Moreover, for inspectors the fact that young people were vulnerable was an important argument for preventive and early intervention, and an argument against waiting for them to help themselves.

## Discussion

In this paper, we analyzed how inspectors include the perspectives of adolescents on good care in their assessment of health and social care services. The themes on quality of care, which we identified from the interviews and focus groups the inspectors held with adolescents are congruent with research on young people’s preferences in quality of care [44, 45]. Inspectors and adolescents agree upon the importance of timely care, opportunities for personal development and a respectful relationship. Yet, their views on quality of care clash with regard to sharing information, creating solutions and the moment to offer help and assistance.

We identified three ways that inspectors dealt with the clashes between their own views and those of service users. First, inspectors place more value on their own views. Following Mol [46], we call this way of dealing with the tension ‘creating a hierarchy’. Establishing a hierarchy creates an order for differing perspectives, which reduces discrepancies as one perspective is made to win. This facilitates decisions on how to act, while discrepancies continue to exist [46]. This dealing mechanism fits neatly in the regulatory context as in the assessment phase of the inspection process inspectors must often balance various views to decide whether the services under scrutiny meet the inspection criteria [33, 47, 48].

A second strategy is passing the tension onto others, in this case professionals. Inspectors state that the professionals providing care to young people should be able to act according to the expectations of both inspectors and adolescents. According to inspectors, professionals should weigh all considerations and make decisions that are appropriate to the specific situation. This requires a situational judgment in which inspectors look closely into the considerations of professionals and discuss, rather than merely assess, what good care entails in a specific situation [49].

A third strategy is separating the conflicting perspectives. For inspectors way of dealing with tensions opens up the opportunity to use adolescents’ information, while still applying the inspection criteria that conflict with this information. This strategy is seen in other inspectorates as well. For example, the English Care Quality Commission adds the perspective of young people and other service users in a separate section of their inspection reports [50].

In our data, while these three strategies limit the influence of the adolescents when their views conflict with the inspectors’ perspective, they do not limit or enhance the influence of service users in themselves. The result of the first, creating a hierarchy, could potentially lead to inspectors prioritizing the view of adolescents, setting their own view aside. The second strategy passes the tension to professionals who may incorporate the views of adolescents in their decision on what to do. The third strategy describes the adolescents’ perspective separately, which may draw extra attention to their voices.

The main reason to engage service users in the inspection process is that they express a distinct perspective on what quality of care is [9, 10, 16, 17]. Inspectors do use adolescents’ views in their reports; they used adolescents’ information to substantiate and illustrate their view (when the perspectives were similar) and they used the information separately from the inspectors’ views (when their views differed). However, our data did not include examples of inspectors changing their opinions based on the views of adolescents. We offer three explanations.

First, part of the explanation is related to the characteristics of the adolescents involved in the inspection [2, 12, 14], in this case their vulnerability. Inspectors consider these adolescents as members of a vulnerable group requiring protection. They tap into the widespread assumption that vulnerable people are in need of special treatment and that intervening in their lives is permitted [51]. Although they may acknowledge what the adolescents think, inspectors believe that they know what is best for this group. Hence, inspectors will not set their own standards and criteria aside.

Second, the explanation is related to the organization where participation takes place, specifically organizational rules and routines [6, 11, 12, 14]. In our case, the existing inspection criteria steered the inspection process [52]. These criteria were already set before adolescents were involved. The criteria turned out to be solid and not easy to change by anyone else than inspectors.

Although the literature relates the difficulties of involving service users mainly to participants and the organization where participation takes place, we add the external context as a third explanation. A fundamental tenet of policy in the Netherlands is that it is better to prevent than to solve problems [53–55]. Investigations into the death of abused or seriously injured people have criticized professionals and care organizations for providing fragmented services, not sharing essential information and not intervening earlier [56–58]. The critique also included inspectorates who were criticized for responding too late to important signs of poor service [20]. This criticism has had an important impact on public confidence in the accountability and legitimacy of inspectorates [7, 20, 56]. As a consequence of this external critique, inspectorates have placed greater emphasis on prevention and early intervention. Active citizenship and prevention are both part of Dutch youth policy [53]. However, in this case, the value of prevention is so dominant that any input from adolescents that goes against this value is put aside. For inspectors, the external context cannot be easily disregarded and limits their room to allow the voice of adolescents influence their decision-making. Consequently, service user involvement cannot reach its full potential.

## Conclusions

Service user involvement in inspections potentially impacts the quality of care. Yet, conflicts between the views of service users and inspectors are not easily overcome in the regulatory context. We offer two suggestions to make the involvement of service users more meaningful.

Firstly, inspectors may involve service users (and other stakeholders) in the development of inspection criteria. When criteria have not yet been set, including service users’ perspectives allows inspectors to discuss various views to form their opinion and prioritize criteria in the dialogue with others. Following up this suggestion, JISD inspectors are currently experimenting with the involvement of service users in the development of new inspection criteria for vulnerable families with multiple problems, which may be a subject for further study. As we found that the perspective of inspectors cannot always be changed (in situations determined by the external context), it is important that inspectors make the values underpinning their views on good care more explicit.

Secondly, inspectors should allow a situational judgment, discussing the specificities of a situation and applying their inspection criteria more flexibly. A concrete example of this suggestion is value-based inspections, which holds the values and principles underlying decisions central [59]. This would mean that in one situation inspectors could decide that privacy must prevail over the exchange of information between professionals, while in another situation sharing information would have priority. Service users and other stakeholders could be part of these discussions.

## Abbreviations

JISD: Joint inspectorate social domain
